# Nipah Virus in Focus: A Comprehensive Review of the Pathogenesis, Epidemiological Patterns, Diagnostic Advances, and Future Public Health Strategies

**DOI:** 10.7759/cureus.103807

**Published:** 2026-02-17

**Authors:** Swathi Gurajala, Sai Saranya Gurajala

**Affiliations:** 1 Department of Respiratory Care, College of Applied Medical Sciences, Imam Abdul Rahman Bin Faisal University, Jubail, SAU; 2 Department of Biology, International Indian School, Jubail, SAU

**Keywords:** antiviral therapeutics, epidemiology, henipavirus, molecular diagnostics, nipah virus, one health, pathogenesis, point‑of‑care testing, vaccine development, zoonotic infections

## Abstract

Nipah virus (NiV) remains one of the most concerning emerging pathogens due to its repeated spillover events, high mortality, and the absence of licensed vaccines or treatments. Its recurrence demonstrates that the ecological factors facilitating such outbreaks, including those linked to bat hosts, are still present. These patterns highlight the continued need for a coordinated One Health approach that connects human, animal, and environmental health.

This review has four aims: (1) to summarize current understanding of NiV biology and pathogenesis, (2) to describe the epidemiological trends that shape how outbreaks unfold, (3) to assess recent advances in diagnostics, and (4) to highlight emerging strategies for prevention and control.

Our narrative synthesis shows that NiV’s ability to infect both the respiratory and nervous systems, combined with strong immune‑evasion mechanisms, contributes to rapid deterioration in many patients. Epidemiological evidence from South and Southeast Asia consistently links outbreaks to bat reservoirs, occasional intermediate hosts, and increasingly recognized person‑to‑person transmission. In diagnostics, improvements in rapid molecular tests, point‑of‑care tools, and serological platforms have expanded the ability to detect cases quickly in field settings. Promising progress is also being made in surveillance systems, ecological risk assessment, and the development of antiviral agents and vaccine candidates.

By synthesizing current scientific and public health insights, this review underscores the need for sustained multidisciplinary strategies to mitigate future NiV threats.

## Introduction and background

The emergence of the Nipah virus (NiV) as a significant threat to global health security represents a quintessential challenge in the management of zoonotic infectious diseases. Classified as a member of the genus *Henipavirus *within the family Paramyxoviridae, NiV is a highly virulent emerging zoonotic pathogen with substantial epidemic and pandemic potential, characterized by its capacity for both animal-to-human and human-to-human transmission [1]. Since its initial discovery in an outbreak in Malaysia in 1998, this virus has caused recurrent outbreaks across South and Southeast Asia, consistently exhibiting high case-fatality rates (CFR) [2]. Recurrent outbreaks, including those recently reported in Kerala, India, in 2018, 2021, and 2023, highlight the continued risk of transmission and underscore the need for integrated surveillance and rapid response systems [2]. The virus is maintained in nature by fruit bats of the *Pteropus *genus, which act as its primary reservoir, with transmission to humans occurring either directly from bats, via contaminated food sources such as date palm sap, or indirectly through intermediate amplifier hosts such as pigs [3]. NiV infection in humans is characterized by a wide spectrum of clinical manifestations ranging from mild febrile illness to severe acute encephalitis and respiratory failure, with reported case‑fatality rates varying from 40% to as high as 90% depending on the outbreak and region. The virus exhibits marked neurotropism and pneumotropism, with pathogenesis driven by endothelial damage, leukocyte invasion, systemic vasculitis, and immune evasion mechanisms that enable widespread organ involvement [1].

Despite its high mortality and growing geographical footprint, no licensed antiviral treatment or human vaccine currently exists for NiV [4]. Clinical management remains largely supportive, though multiple experimental therapeutics, including monoclonal antibodies and antiviral agents [4]. Advances in molecular diagnostics and point‑of‑care detection strategies have improved outbreak detection capabilities; however, gaps persist in early recognition and field applicability, particularly in resource‑limited settings. The complex interplay between the virus's molecular biology, the ecology of its natural reservoir, fruit bats of the genus *Pteropus*, and the socio-economic factors driving human-animal interactions necessitates a multidisciplinary approach to surveillance, diagnostics, and drug development [3]. 

Given its high pathogenicity, evolving transmission dynamics, and significant global health implications, NiV remains a priority pathogen for the World Health Organization (WHO) [4]. A comprehensive synthesis of its pathogenesis, epidemiological trends, diagnostic innovations, and public health strategies is therefore essential for strengthening preparedness frameworks and guiding future research and policy initiatives.

## Review

Molecular architecture and genomic organization

The NiV is an enveloped, negative-sense, single-stranded RNA virus with a non-segmented genome spanning approximately 18.2 Kb [2]. Its structural morphology is pleomorphic, varying in shape from spherical to filamentous, with particles ranging in size from 40 nm to 1900 nm [4]. The viral envelope is derived from the host cell membrane during the budding process and is embedded with two essential surface glycoproteins: the attachment glycoprotein (G) and the fusion protein (F) [2].

The genomic structure comprises six major genes arranged in a 3’-5’ orientation, encoding the nucleocapsid (N) protein, phosphoprotein (P), matrix (M) protein, F protein, G glycoprotein, and the large (L) protein or RNA-dependent RNA polymerase. The N protein encapsulates the viral RNA to form the ribonucleoprotein (RNP) complex, which serves as the template for transcription and replication. The P gene is particularly notable for its coding complexity; through alternative start codons and ribosomal shunting, it produces not only the P protein but also three non-structural proteins: C, V, and W [2]. These non-structural proteins are critical determinants of pathogenesis, primarily functioning as potent inhibitors of host interferon (IFN) signaling, thereby disabling the host’s innate immune response [5,6].

The M protein plays a central role in viral assembly and budding, coordinating the interaction between the RNP complex and the surface glycoproteins at the host cell membrane [7]. The G glycoprotein is a type II membrane protein that exists as tetramers consisting of disulfide-linked dimers on the viral envelope. The G protein facilitates entry by binding to highly conserved cellular receptors, specifically ephrin B2 and ephrin B3. This binding event triggers a conformational change in the F protein, which is a type I membrane protein, ultimately mediating the fusion of the viral and cellular membranes [2].

The genetic diversity of the virus is categorized into two distinct lineages: the Malaysia genotype (NiV-M) and the Bangladesh genotype (NiV-B), which share roughly 91.8% nucleotide identity but exhibit notable differences in virulence and transmission patterns [8]. Table 1 summarizes the functions of the various proteins in the virus.

**Table 1:** Functional Roles of Nipah Virus' Structural and Accessory Proteins NS: Non-structural Source: [2]

Protein	Genetic Category	Primary Biological Function	Impact on Pathogenesis
Nucleoprotein (N)	Structural	Encapsidates the RNA genome to form the RNP complex	Protects viral RNA and serves as a template for replication.
Phosphoprotein (P)	Structural	Acts as a cofactor for the RNA-dependent RNA polymerase (L)	Essential for RNA synthesis and RNP complex stability.
Matrix protein (M)	Structural	Orchestrates viral assembly and budding at the plasma membrane	Determines the structural integrity and exit of new virions.
Fusion protein (F)	Structural	Mediates membrane fusion between viral and host envelopes	Critical for host cell entry and the formation of syncytia.
Glycoprotein (G)	Structural	Binds to ephrin B2/B3 receptors on host cells	Dictates tissue tropism and is the primary target for neutralization.
Polymerase (L)	Structural	Catalyzes RNA transcription and genome replication	Large protein responsible for the enzymatic activity of replication.
C, V, W proteins	Accessory/NS	Antagonize interferon (IFN) signaling	Facilitates systemic spread by evading innate immune detection.

Mechanism of viral entry and pathogenesis

The pathogenesis of NiV is defined by its broad tissue tropism and its ability to cause systemic vasculitis and severe neurological damage [9]. The primary entry point into the host is typically the oro-nasopharyngeal route, where the virus initiates replication in the respiratory epithelium [2]. The utilization of ephrin B2 and ephrin B3 as entry receptors is the fundamental driver of NiV’s diverse cellular targets [10]. 

Leukocytes contribute to viral dissemination and inflammation. NiV-loaded leukocytes can transfer infection to permissive cells and migrate across endothelial barriers, facilitating CNS entry and systemic spread. In lung tissue specifically, infiltrating leukocytes amplify inflammation, cytokine production, and oxidative stress, worsening tissue damage [2].

Receptor interaction and tissue tropism

Ephrin B2 is an evolutionarily conserved protein involved in vascular development and is expressed on the surface of endothelial cells, smooth muscle cells, and neurons [11]. Ephrin B3, while sharing homology with ephrin B2, has a more specialized role in the central nervous system (CNS), particularly in the brainstem and spinal cord. NiV-G binds to ephrin B2 with extremely high affinity, characterized by a dissociation constant (Kd) of approximately 0.27nM. While it also binds to ephrin B3 with high affinity (Kd approx. 0.78nM), the interaction is roughly 30-fold more potent than that of its close relative, the Hendra virus, which may explain the distinct neurological presentation and high lethality of Nipah infections [10].

The distribution of these receptors explains the hallmark features of the disease. Infection of the endothelial cells lining the small blood vessels and capillaries leads to widespread vasculitis, thrombosis, and parenchymal necrosis. In the lungs, NiV targets the bronchial and alveolar epithelial cells as well as the pulmonary endothelial cells, resulting in severe respiratory distress, hemorrhage, and the formation of syncytia [9].

Systemic dissemination and immune evasion

The "Trojan horse" model of dissemination suggests that NiV can adhere to circulating leukocytes, such as lymphocytes and monocytes, without necessarily infecting them productively. These leukocytes act as vehicles, transporting the virus through the bloodstream to various organs, including the spleen, kidneys, and brain [2]. The disruption of the blood-brain barrier (BBB) is a critical step in the development of encephalitis. NiV may cross the BBB through the direct infection of microvascular endothelial cells or by entering the CNS via the olfactory nerve, bypassing the traditional hematogenous route [9].

To establish a successful infection, NiV must overcome the host's innate antiviral defenses. The non-structural V and W proteins play a crucial role in this process, targeting the IFN signaling pathway. The V protein inhibits the induction of IFN-alpha and IFN -beta by blocking the phosphorylation and subsequent nuclear translocation of STAT1 and STAT2 proteins. Similarly, the W protein can sequester STAT1 in the nucleus, effectively silencing the transcription of IFN-stimulated genes (ISGs) [2]. This suppression of the innate immune response allows for rapid viral replication and systemic spread before the adaptive immune system can mount an effective defense.

Comparative pathogenesis of NiV strains

The epidemiological data reveal significant differences between the NiV-M and NiV-B lineages. These differences are not merely geographical but extend to the clinical manifestation and transmission dynamics of the virus [12]. Table 2 lists the differences between NiV-M and NiV-B virus lineages.

**Table 2:** Comparative Epidemiology of Nipah Virus Clades CFR: Case-Fatality Rates Sources: [6,12,13]

Characteristic	Malaysia Lineage (NiV-M)	Bangladesh Lineage (NiV-B)
Intermediate host involvement	Essential (pigs)	Occasional/None (direct spillover common)
Transmission seasonality	Non-seasonal (occupational)	Winter season (palm sap harvest)
Human-to-human spread	Limited/None	Frequent
Mortality rate (CFR)	~40%	>70%
Clinical presentation	Primarily encephalitis	Respiratory distress + Encephalitis
Replication kinetics	Rapid cytopathology in vitro	Delayed progression in vivo
Incubation period	Nine to 14 days	Approximately nine days
Respiratory involvement	Moderate	Severe/ Acute respiratory distress syndrome frequent
Reservoir species	*Pteropus vampyrus*, *Pteropus hypomelanus *	*Pteropus medius *

While NiV-M showed high cytopathogenicity in early laboratory models, causing extensive syncytia formation and rapid cell death, NiV-B is characterized by more severe respiratory involvement, with cough and respiratory distress reported in up to 75% of cases compared to only 14% for NiV-M [13]. In non-human primate models, NiV-B infection often results in lung lobes that are more engorged with edema, reflecting the clinical presentation of life-threatening acute respiratory distress syndrome (ARDS) observed in human outbreaks in Bangladesh and India [6].

Ecological reservoirs and zoonotic transmission

The persistence of NiV in the environment is inextricably linked to the ecology of *Pteropid *fruit bats, also known as flying foxes. These bats serve as the natural reservoir, carrying the virus without showing signs of clinical illness [1]. Understanding the factors that precipitate the spread of the virus from bats to humans is essential for developing preventive strategies.

Bat ecology and viral shedding

*Pteropus *bats are distributed across a wide geographic range, spanning from South Asia to Australia and parts of Africa. They are highly social, nomadic animals that move between roost sites in search of food, which primarily consists of nectar and fruit. Viral shedding in bat populations is often sporadic and may be influenced by seasonal factors, such as the breeding season or periods of nutritional stress [3].

Research into Hendra virus, a closely related *Henipavirus*, has shown that "population fission," the breaking up of large bat colonies into smaller groups, often occurs during food shortages. These smaller groups tend to roost closer to human settlements and agricultural areas, where they may feed on anthropogenic food sources [14]. This behavioral shift increases the likelihood of contact between bats, domestic animals (such as pigs or horses), and humans [15]. 

These bats maintain the virus asymptomatically, shedding it periodically through urine, saliva, feces, and birthing fluids. Environmental survival of the virus is a critical factor in spillover events; studies have shown that NiV can persist for three to seven days in fruit juices or simulated date palm sap at 22 °C, with a half-life of 18 hours in bat urine. The virus is stable between pH 4.0 and 10.0 but is sensitive to common soaps, detergents, and heat, with complete inactivation occurring at 100 °C for more than 15 minutes [15].

Transmission to humans occurs through three primary pathways: direct contact with infected animals, consumption of contaminated food, and human-to-human transmission. In the Malaysian context, pigs served as a critical intermediate host, becoming infected by consuming fruit contaminated by bats and subsequently transmitting the virus to humans via respiratory droplets or direct contact [8]. In the South Asian context (Bangladesh and India), spillover often occurs through the consumption of raw date palm sap contaminated with bat excreta or fruit partially eaten by bats [16].

Human-to-human transmission is a hallmark of the NiV-B clade and is driven by close contact with infected respiratory secretions or body fluids. This is particularly prevalent in healthcare settings, where inadequate infection control can lead to large nosocomial clusters [8]. Historical data indicate that approximately 75% of cases in some outbreaks were linked to hospital exposure [17].

Anthropogenic and climatic influences

Deforestation has significantly impacted bat habitats. In Bangladesh, for instance, forest cover has decreased from 40% to less than 10%, forcing bats to adapt to human-dominated landscapes [18]. This proximity is a major driver of spillover. The consumption of raw date palm sap is a primary transmission route in Bangladesh and India. Bats visit the trees at night to drink the sap being collected in clay pots, contaminating it with their urine or saliva [1].

Climate change is projected to further alter the distribution of *Henipavirus *reservoirs. Ecological niche modeling suggests that by mid-century, a significant increase in suitable habitat for *Pteropus *bats will occur in parts of western Africa, western India, and northern Australia [18]. This expansion of the reservoir's range could introduce the virus to new populations, highlighting the need for expanded surveillance and diagnostic capacity in these regions [19].

Outbreak chronology (1998-2026)

The history of NiV outbreaks reflects a pattern of geographic expansion and evolving transmission dynamics. From the initial agricultural crisis in Malaysia to the recurrent, highly fatal outbreaks in the "Nipah belt" of South Asia, each event has provided critical insights into the virus's potential for human-to-human transmission.

The Malaysia and Singapore Outbreak (1998-1999)

The first recognized outbreak occurred among pig farmers and abattoir workers in Malaysia and Singapore [5]. The virus was initially mistaken for Japanese encephalitis, which delayed the implementation of appropriate control measures. The outbreak was eventually contained through the culling of over one million pigs and the implementation of strict movement restrictions. This event established the role of pigs as intermediate amplifying hosts, where the virus caused severe respiratory disease known as "one-mile cough" or "barking pig syndrome" [1].

Recurrent Outbreaks in Bangladesh and India (2001-2023)

Since 2001, outbreaks have been reported almost annually in Bangladesh, with cases often clustered during the winter months (December to March), coinciding with the date palm sap collection season. In India, the first outbreak was recorded in Siliguri, West Bengal, in 2001, where significant human-to-human transmission occurred in a hospital setting [1].

Kerala has emerged as a particularly high-risk area in India. The state experienced its first outbreak in 2018 in the Kozhikode district, followed by subsequent events in 2019, 2021, and 2023 [5]. The 2018 outbreak was notable for its high CFR (91%) and the identification of the index case as having contracted the virus from an infected baby bat [1]. The 2023 outbreak in Kozhikode necessitated the declaration of containment zones and the testing of hundreds of contacts, demonstrating the state's robust but strained public health infrastructure [20].

The 2024-2026 Crisis and Regional Response

The period leading into early 2026 has been marked by continued viral activity and a tightening of global health security measures. In 2024, Bangladesh reported five laboratory-confirmed fatal cases across various districts [21]. In July 2024, a new infection in Kerala resulted in the death of a 14-year-old boy, prompting renewed concern over the localized endemicity of the virus [5].

Between May and July 2025, a cluster was reported in Kerala's Malappuram and Palakkad districts [5]. A fatal case in Malappuram in May 2025 prompted mass fever surveillance of 3,868 households, demonstrating the intensity of the regional response. The Palakkad cases were significant as they marked the first-ever reported outbreak in that specific district. One adult woman developed symptoms on June 25, 2025, and remained in critical condition on ventilator support for an extended period, while another adult male died on July 12, 2025. Investigation into these cases suggested independent spillover events from fruit bats rather than a single connected chain of transmission [22]. 

In late December 2025 and January 2026, West Bengal reported a new cluster of infections. As of January 27, 2026, two cases were confirmed by the National Centre for Disease Control (NCDC), with 196 contacts traced and found asymptomatic [23]. However, the report of confirmed cases in the state triggered a wave of international concern. Airports in Thailand, Taiwan, and Nepal reintroduced health screenings and travel advisories for passengers arriving from affected regions. Taiwan went as far as proposing to classify Nipah virus infection as a 'Category 5' notifiable disease, the highest alert level, reflecting the perceived risk of rapid transboundary spread [24]. By late January 2026, over 100 close contacts were quarantined in West Bengal as investigators worked to identify the index patient and potential secondary infections [25].

Clinical manifestations and pathological spectrum

NiV infection presents a diverse clinical spectrum, from asymptomatic cases to fatal encephalitis and respiratory failure. Symptoms typically manifest after an incubation period of four to 14 days, although rare instances of incubation up to 45 days have been documented. The illness generally begins with an acute prodromal phase characterized by fever, headache, myalgia, vomiting, and sore throat [5].

As the disease progresses, it frequently involves the respiratory system, leading to cough and breathlessness that may rapidly evolve into acute respiratory distress syndrome (ARDS) or atypical pneumonia. Neurological involvement is common and severe; initial dizziness and drowsiness can progress to acute encephalitis, seizures, and coma within 24 to 48 hours. Survivors often face long-term neurological sequelae, including seizure disorders and personality changes. Notably, the virus can persist in the central nervous system, leading to relapsed or late-onset encephalitis months or even years after the initial infection [5]. 

Table 3 lists the key signs and symptoms and related complications of NiV infection.

**Table 3:** Clinical Progression and Major Complications of Nipah Virus Infection ARDS: Acute Respiratory Distress Syndrome Source: [5]

Phase of Illness	Key Signs and Symptoms	Pathological Complications	Recovery/Long-Term Outcomes
Prodromal	High fever, severe headache, myalgia, sore throat	Non-specific; often misdiagnosed initially.	Most individuals either progress to more severe phases or recover fully if the infection remains subclinical. Asymptomatic or mild cases can occur.
Respiratory	Cough, dyspnea, tachypnea, hypoxia	Atypical pneumonia; ARDS; Respiratory failure.	Survivors of respiratory‑predominant disease generally recover without long‑term pulmonary deficits, though fatigue may persist in some.
Neurological	Confusion, altered mental status, drowsiness	Acute encephalitis; Brain swelling; Focal seizures.	Neurological sequelae occur in ~20–24% of survivors, including behavioral changes, memory issues, motor deficits, and seizure disorders. Late‑onset encephalitis can develop months to years later in ~10%.
Critical	Seizures, coma, cardiovascular collapse	Multi-organ failure; Septicemia; Renal impairment.	Survivors of critical disease may have a significant risk of permanent neurological injury. WHO reports that most survivors make a full recovery, but ~1 in 5 develop chronic neurological complications
Chronic/Late	Seizure disorders; Personality changes	Relapsed encephalitis; Delayed-onset brain inflammation.	Late‑onset or relapsing encephalitis occurs in ~10% of recovered patients. Up to 20% experience persistent long‑term neurological issues such as cognitive impairment, fatigue, mood changes, or focal deficits.

Laboratory diagnostics and biosafety

The high lethality and contagious nature of NiV require diagnostics that are both highly accurate and safe to perform. By 2026, the diagnostic landscape will have seen a shift toward decentralized testing, although BSL-4 containment will remain the standard for handling live virus [5].

Molecular diagnostic technologies

Reverse transcription polymerase chain reaction (RT-PCR) remains the gold standard for diagnosing acute infection, with viral RNA detectable in respiratory secretions, urine, blood, and cerebrospinal fluid (CSF) for approximately two weeks post onset [5]. However, traditional PCR requires sophisticated laboratory infrastructure and trained personnel, which are often unavailable in remote endemic areas [26].

To address these gaps, several isothermal amplification methods have been developed: 1. Recombinase polymerase amplification (RPA) and recombinase adenoviral amplification (RAA): These tests operate at a constant temperature (typically 37-42 °C) and can be paired with lateral flow strips for a simple visual read-out. They have demonstrated analytical sensitivity as low as 1,000 copies per microliter [7]. 2. RT-loop-mediated isothermal amplification (LAMP): This method uses four to six primers to achieve high specificity and sensitivity. RT-LAMP assays have been developed that effectively detect all known strains of NiV, including the Malaysian, Bangladeshi, and Thai bat isolates [7]. 3. CRISPR-based diagnostics: The use of CRISPR/Cas12a systems in combination with RAA has shown promise for highly sensitive detection, with some assays reaching a limit of detection (LOD) of 10 copies per microliter [7].

In 2025, a landmark study described an RT-RAA-CRISPR/Cas13a detection method that achieved high sensitivity (1.565 copies/uL) and specificity for NiV within 80 minutes. This platform utilizes lateral flow strips (LFS) for visual readout, providing a potentially transformative tool for rapid onsite detection in remote "Nipah belt" regions [27].

Serology and surveillance

Serological assays, such as enzyme-linked immunosorbent assay (ELISA) for IgM and IgG, are crucial for retrospective diagnosis and longitudinal studies [5]. IgM antibodies typically peak around 20 days post-exposure and can persist for up to a month, while IgG antibodies can remain detectable for over a year [7]. By 2024, manufacturers like Abbott and Alpha Diagnostic International expanded the availability of recombinant antigens and ELISA kits to support global research and validation efforts [28].

Table 4 has the list of diagnostic tests available for NiV.

**Table 4:** Nipah Virus Diagnostic Assays and Parameters RT-qPCR: Real Time Quantitative Polymerase Chain Reaction; ELISA: Enzyme-Linked Immunosorbent Assay; RPA: Recombinase Polymerase Amplification; LFA: Lateral Flow Assay Sources: [7,28]

Assay Platform	Biological Target	Primary Use Case	Sensitivity/Specificity	Turnaround Time
RT-qPCR	Viral RNA (N, L genes)	Gold standard for acute case confirmation	High/High	4-24 hours
IgM ELISA	Host IgM antibodies	Outbreak investigation and active infection	Moderate/High	4-6 hours
IgG ELISA	Host IgG antibodies	Epidemiological and retrospective surveillance	High/Moderate	4-6 hours
CRISPR-Cas13a	Viral RNA	Rapid point-of-care screening	Ultra-high/High	< 90 minutes
RPA / LFA	Viral RNA	Low-resource field settings	High/Moderate	30 - 60 minutes
Virus isolation	Live infectious virus	Definitive reference laboratory characterization	High/Absolute	3-7 days

Biosafety protocols and sample inactivation

Working with live NiV is restricted to BSL-4 laboratories, but clinical samples can be processed in lower-containment settings if they are properly inactivated [5]. In January 2026, research validated several chemical and physical inactivation protocols, which are as follows: Chemical Agents: 10% neutral buffered formalin and TRIzol reagent were shown to reduce infectious particles to undetectable levels in tissues and serum samples from non-human primates [29]. Thermal and UV Inactivation: Heating samples at 60 °C for 30 minutes, or at 56 °C for 60 minutes, successfully inactivated NiV in serum.UV-C irradiation for 30 minutes was also found to be effective, although excess protein in serum can interfere with the process, suggesting that combined protocols are safer [30].

Infection prevention and control (IPC) in clinical settings

Effective IPC is the primary defense against the amplification of Nipah outbreaks in healthcare facilities. The core of NiV IPC is the implementation of transmission-based precautions, specifically contact, droplet, and airborne measures [5]. Table 5 lists the recommended IPC practices based on the patient's clinical condition.

**Table 5:** Usage of PPE and Patient Placement Guidelines based on Clinical Stability PPE: Personal Protective Equipment; ARDS: Acute Respiratory Distress Syndrome; AIIR: Airborne Infection Isolation Room; FFP-3: Filtering Face Piece-3; PAPR: Powered Air-Purifying Respirator; VHF: Viral Hemorrhagic Fever Source: [5]

Clinical Status of Suspect/Confirmed Case	Recommended Isolation Facility	Required PPE
Clinically stable (no vomiting/hemorrhage)	AIIR or individual room	Gown, gloves, eye protection, N95/FFP3 respirator
Clinically unstable (vomiting/ARDS)	AIIR (negative pressure)	Fluid-resistant coveralls, double/triple gloves, apron, PAPR or FFP3
Aerosol-generating procedures (AGP)	AIIR	Impermeable gown, PAPR, face shield, shoe covers
Post-mortem handling	Specialized isolation area	PPE as per clinically unstable VHF guidance

Environmental and waste management protocols

Environmental cleaning is vital as NiV remains viable on surfaces if not properly disinfected. Bleach-based disinfectants are recommended for terminal cleaning of rooms, with UV-C or hydrogen peroxide vaporization as optional enhancements. Healthcare workers must use complete disposable personal protective equipment (PPE) during sample collection, including double surgical gloves and N95 masks. Waste generated during care is considered Category A infectious waste and must be handled according to strict biomedical waste guidelines, often involving incineration [5].

The "Buddy" system is mandatory for high-risk clinical environments. A trained assistant (buddy) must oversee the donning and doffing process in designated "Amber" and "Green" zones to ensure no breaches in protocol occur. Doffing is identified as the highest-risk phase for healthcare worker exposure, requiring slow, deliberate movements and constant hand hygiene between steps [31].

Therapeutic strategies and the pharmaceutical pipeline

The absence of an approved treatment for NiV remains a critical vulnerability. Current clinical management is supportive, focusing on the treatment of secondary infections and neurological complications [5].

Monoclonal Antibodies (mAbs)

mAbs represent the most advanced therapeutic category. The human mAB m102.4 targets the receptor-binding domain of the G glycoprotein. Since 2010, m102.4 has been administered to 18 patients globally on an emergency-use or compassionate-use basis. MBP1F5 (1F5), a human mAb specific to the fusion (F) glycoprotein, has shown a 100% success rate in animal models, even when given five days post infection [24]. Unlike the earlier m102.4 mAb, which targets the attachment glycoprotein, 1F5's focus on the fusion protein provides immediate neutralization and prevents viral entry. It has completed Phase I clinical trials, which assessed its safety, tolerability, and pharmacokinetics in healthy adults [32]. In 2025, the Indian Council of Medical Research (ICMR) actively sought industry partnerships to develop indigenous mAb therapies, recognizing the need for sustainable local production during outbreaks [33].

Experimental Antivirals

Several small-molecule antivirals have shown potential.

VV116: This oral nucleoside analog, already approved for COVID-19 in China and Uzbekistan, has shown broad-spectrum activity against multiple RNA viruses. A study published in November 2025 in Emerging Microbes & Infections demonstrated that VV116 inhibited NiV replication in golden hamsters, achieving 100% survival in high-dose groups. Researchers noted that the drug significantly reduced viral loads in the lungs, spleen, and brain [34].

Remdesivir and favipiravir: These drugs have shown promise in preclinical studies and limited clinical use, although large-scale efficacy data for NiV are lacking [35].

Vaccines 

The pursuit of a NiV vaccine is a centerpiece of the WHO R&D Blueprint and the Coalition for Epidemic Preparedness Innovations (CEPI) strategy [36]. Because outbreaks are sporadic and involve small numbers of people, traditional Phase III efficacy trials are nearly impossible to conduct. One modeling study estimated that a mass vaccination trial in Bangladesh would take 43 years to reach statistical significance. Consequently, vaccine licensure will likely depend on the "Animal Rule" demonstrating efficacy in well-characterized animal models and safety/immunogenicity in human Phase I and II trials [37].

ChAdOx1 NipahB: The Phase II Milestone

In early December 2025, the University of Oxford launched the world’s first Phase II clinical trial for a Nipah vaccine candidate, ChAdOx1 NipahB. This vaccine uses the same chimpanzee adenoviral vector platform as the successful Oxford-AstraZeneca COVID-19 vaccine. The trial is being conducted in Bangladesh in collaboration with the International Centre for Diarrhoeal Disease Research, Bangladesh (icddr,b), and is funded by CEPI. It aims to enroll 306 healthy participants (ages 18 to 55) to evaluate the vaccine's safety and the strength of the immune response in a population where the virus is endemic. The vaccine was manufactured for the trial by the Serum Institute of India (SII), demonstrating the strength of South-South and North-South research partnerships [4].

The Global Vaccine Reserve

Parallel to the Phase II trials, CEPI, Oxford, and SII announced in October 2025 the establishment of the world’s largest investigational ready reserve of a Nipah vaccine. Supported by $7.3 million in funding from CEPI, SII is manufacturing a reserve of up to 100,000 doses to be stored in Pune, India. These doses are intended for emergency deployment during future outbreaks to generate real-world data and potentially halt the spread of the virus in its tracks [38].

Table 6 lists the current vaccines in clinical trials for NiV prevention.

**Table 6:** Vaccines in Clinical Trials Against Nipah Virus SII: Serum Institute India; CEPI: Coalition for Epidemic Preparedness Innovations; NIAID: National Institute of Allergy and Infectious Diseases Sources: [37,38,39]

Vaccine Candidate	Platform	Status (Jan 2026)	Developer/Funder
ChAdOx1 NipahB	Viral vector (ChAdOx1)	Phase II clinical trial	Oxford/SII/CEPI
mRNA-1215	mRNA	Phase I	Moderna/NIAID
rVSV-Nipah (PHV02)	Viral vector (VSV)	Phase I	Public health vaccines/NIAID
HeV-sG-V	Recombinant protein	Phase I	Various

Global health security frameworks and the One Health Paradigm

The management of NiV is now recognized as a vital component of global health security. The virus’s potential for mutation and its high lethality place it in the same category of threat as "Disease X" [39].

One Health and Surveillance Roadmaps

The WHO South-East Asia Region (SEAR) Strategic Roadmap for Health Security (2023-2027) emphasizes the "One Health" approach, which integrates human, animal, and environmental surveillance [19]. This includes monitoring viral circulation in *Pteropus* bats and agricultural livestock to identify high-risk spillover interfaces [40]. The roadmap calls for enhanced laboratory capacity in regional hubs and the standardization of clinical management protocols [19].

Chemical, Biological, Radiological, Nuclear, and Explosive (CBRNE) Strategies and Biocontainment

The use of CBRNE frameworks for outbreak containment has been proposed by military and civilian security analysts. This model suggests the rapid deployment of mobile isolation pods and the use of aerosolized disinfectant systems in healthcare settings to protect frontline workers. The 2025-2026 response to the West Bengal cluster showcased the beginning of such unified architectures, with rapid contact tracing and stringent hospital alerts issued across multiple districts [20].

The CEPI 100 Days Mission

The CEPI strategic agenda (CEPI 2.0 and 3.0) revolves around the 100 Days Mission, a goal to deliver safe and effective vaccines within 100 days of identifying a new pathogen or outbreak. For the Nipah virus, this mission is being realized through the creation of "vaccine libraries", collections of candidate vaccines for various virus families, and the establishment of scalable manufacturing networks in low- and middle-income countries (LMICs) [39]. The partnership with the SII is a prime example of this strategy, leveraging the world’s largest vaccine manufacturing capacity to support a priority pathogen for the Global South [38].

Future outlook

The trajectory of NiV research and response through 2026 reflects a sophisticated evolution in our understanding of high-threat pathogens. The transition from the reactive measures of 1998 to the proactive clinical trials and dose reserves of 2026 indicates a fundamental shift in global preparedness.

Clinical and Scientific Gaps

Despite the significant milestones, several challenges remain. The clinical performance data for many point-of-care diagnostic prototypes are still limited, and the cost of near-point-of-care molecular instruments may be a barrier in rural endemic areas [7]. Furthermore, the lack of a licensed vaccine or therapeutic means that the primary defense remains non-pharmaceutical interventions: avoiding raw date palm sap, implementing rigorous infection control in hospitals, and managing deceased bodies with extreme caution [5].

Ecological Resilience

The high seroprevalence (13.92%) found in bat populations in the Philippines suggests that the geographic extent of the virus is far wider than current outbreak maps indicate [41]. The long-term mitigation of NiV risk requires addressing the root causes of spillover. This includes protecting bat habitats to reduce the population fission and nutritional stress that lead bats toward human settlements [15]. Public health campaigns aimed at behavioral change, such as the promotion of sap-collection methods that use bamboo skirts to prevent bat access, are essential components of a community-centered response [38].

Strategic recommendations

To sustain the progress made by 2026, the global community must 1) Accelerate regulatory pathways: utilize the European Medicines Agency (EMA)PRIority MEdicines (PRIME) and other expedited review designations to bring Phase II candidates toward licensure using the Animal Rule where human efficacy trials are not feasible [4]. 2) Localize diagnostic production: support the manufacturing of molecular and serological tests within endemic countries like India and Bangladesh to ensure resilient supply chains during outbreaks [28]. Expand One Health surveillance: invest in long-term ecological monitoring of *Pteropus *bat populations to identify changes in viral shedding patterns and migration driven by climate change [19]. Strengthen frontline biocontainment: provide ongoing training and adequate personal protective equipment to healthcare workers in rural clinics, who are often the first to encounter undiagnosed cases [42].

## Conclusions

The NiV remains a formidable biological threat, but the convergence of cutting-edge molecular biology, innovative diagnostic technologies, and robust international cooperation frameworks suggests that the world is better prepared than ever to confront its next emergence. The successful completion of the current Phase II trials will be a defining moment for the Global South, proving that collaborative research can deliver life-saving countermeasures for diseases that were once considered neglected.
